# ﻿Three new species of *Orchestina* Simon, 1882 (Araneae, Oonopidae) from Xizang, China

**DOI:** 10.3897/zookeys.1239.147523

**Published:** 2025-05-30

**Authors:** Yang Zhou, Yanfeng Tong, Dongju Bian, Shuqiang Li

**Affiliations:** 1 College of Life Science, Shenyang Normal University, Shenyang 110034, China Shenyang Normal University Shenyang China; 2 Key Laboratory of Forest Ecology and Management, Institute of Applied Ecology, Chinese Academy of Sciences, Shenyang 110016, China Chinese Academy of Sciences Shenyang China; 3 Institute of Zoology, Chinese Academy of Sciences, Beijing 100101, China Chinese Academy of Sciences Beijing China

**Keywords:** Goblin spiders, new genus record, taxonomy, Tibet

## Abstract

The genus *Orchestina* is recorded for the first time from Xizang, China. One known species and three new species are reported: *Orchestinacolubrina* Liu, Henrard & Xu, 2019 (♂♀), *Orchestinalini* Tong & Li, **sp. nov.** (♂♀), *Orchestinawuzu* Tong & Li, **sp. nov.** (♂♀) and *Orchestinayigong* Tong & Li, **sp. nov.** (♂).

## ﻿Introduction

Oonopidae, also called goblin spiders, are a diverse spider family with 1962 extant described species in 115 genera (WSC 2025). The genus *Orchestina* Simon, 1882 is a species-rich group of oonopid spiders that currently contains 170 extant species (WSC 2025). So far, 21 species of this genus are known to occur in China (Tong and Li 2011; Liu et al. 2016, 2019; Lin et al. 2024; Song et al. 2024; Wang et al. 2024a).

Xizang, also called Tibet, is the main part of the Qinghai-Tibet Plateau, with an area of over 1.2 million square km, and serves as an important ecological security screen. The early research on oonopid spiders in Xizang was very limited, with only two species, i.e., *Gamasomorphalinzhiensis* Hu, 2001, and *Ischnothyreuslinzhiensis* Hu, 2001 recorded in Linzhi, Xizang (Hu 2001). In recent years, one new genus and many species have been documented or described in this region (Cheng et al. 2021; Tong et al. 2023; Wang et al. 2024b; Shi et al. 2025). Currently, there are a total of 5 genera and 15 species of oonopids distributed in Xizang. There are no records of the genus *Orchestina* from Xizang.

While studying oonopid spiders collected from Xizang Autonomous Region, one known species and three new species of the genus *Orchestina* were recognized. It is the first time that this genus has been found in Xizang. The present paper aims to provide detailed descriptions and illustrations of the three new species.

## ﻿Material and methods

Specimens were examined using a Leica M205 C stereomicroscope. Details of body parts and measurements (in millimeters) were studied under an Olympus BX51 compound microscope. Photos were made with a Canon EOS 750D zoom digital camera (18 megapixels) mounted on an Olympus BX51 compound microscope. Raw photos were first stacked with Helicon Focus v. 8.2.0 to get the composite images, which were then processed in Adobe Photoshop CC 2020. The distribution map was generated with ArcGIS v. 10.2 (ESRI Inc.). Terminology and taxonomic descriptions follow Song et al. (2024). Type material is deposited in the Shenyang Normal University (SYNU) in Shenyang, Liaoning Province, China (curator: Yanfeng Tong).

The following abbreviations are used in the text and figures: **ARe** = anterior receptaculum; **AUS** = anterior uterine sclerite; **Ex** = dorsolateral extension; **PME** = posterior median eyes; **Po** = pocket; **PP** = posterior plate; **Pr** = protrusion; **Se** = serrula.

## ﻿Taxonomy

**Family Oonopidae Simon, 1890**

### ﻿Genus Orchestina

Taxon classificationAnimaliaAraneaeOonopidae

Simon, 1882

FE244D2A-D398-5D5F-856D-82854B1567D6

#### Type species.

*Schoenobatespavesii* Simon, 1873.

#### Remark.

*Orchestina* was considered a senior synonym of *Ferchestina* Saaristo & Marusik, 2004 (type *F.storozhenkoi* Saaristo & Marusik, 2004) by Platnick et al. (2012).

### ﻿ Orchestina colubrina

Taxon classificationAnimaliaAraneaeOonopidae

Liu, Henrard & Xu, 2019

96506132-15B6-51D0-AA7E-BC584CC3035B

Orchestina colubrina Liu, Henrard & Xu, in Liu et al. 2019: 246, figs 10A–H, 11A–F (f); Song et al. 2024: 258, figs 2A–F, 3A–I, 7E–F (m, f).

#### Material examined.

China • 1 ♂ (SYNU-F-053), beating bushes; Xizang, Linzhi City, Motuo Co., Beibeng Town (2 km northeast of the road from Beibeng Town to the Motuo Highway); 29°15.048'N, 95°11.514'E, 799 m; 21.VII.2015; J. Wu leg. • 1 ♀ (SYNU-F-054); same data as above • 1 ♂ (SYNU-F-051), beating bushes; Motuo Co., Yarlung Zangbo River near Ximo River Bridge and Lugu Suspension Bridge; 29°21.114'N, 95°20.502'E, 707 m; 25.VIII.2015; J. Wu leg. • 1 ♂ (SYNU-F-052), beating bushes; Motuo Co., Madi Vill., near Deguo Bridge; 29°24.114'N, 95°22.632'E, 837 m; 18.VIII.2015; J. Wu leg.

#### Diagnosis and description.

See Song et al. (2024).

#### Distribution.

China (Jiangxi, Xizang, Yunnan) (Fig. 9).

### ﻿ Orchestina lini

Taxon classificationAnimaliaAraneaeOonopidae

Tong & Li sp. nov.

2C5DBAE0-83FD-5F2C-8888-419264382816

https://zoobank.org/8CADFD9D-732C-4B47-BEAB-EEA3D02C2676

 Figs 1 –3

#### Material examined.

***Holotype*** China • ♂ (SYNU-F-055), sifting forest leaf litter; Xizang, Linzhi City, Motuo Co., near Yadong Vill.; 29°20.605'N, 95°20.807'E, 1360 ± 5 m; 6.VIII.2013; L. Lin leg. ***Paratypes*.** China • 1 ♀ (SYNU-F-089), sifting forest leaf litter; Xizang, Linzhi City, Pome Co., Gangxiang Nature Reserve; 29°51.076'N, 95°35.315'E, 2750 ± 10 m; 31.VII.2013; L. Lin leg. • 1 ♀ (SYNU-F-090), sifting forest leaf litter; Xizang, Linzhi City, Pome Co., Near Zhamu Town; 29°52.205'N, 95°45.894'E, 2700 ± 16 m; 18.VII.2013; L. Lin leg.

#### Etymology.

The specific name is named after the collector, Linghui Lin.

#### Diagnosis.

**Figure 1.**
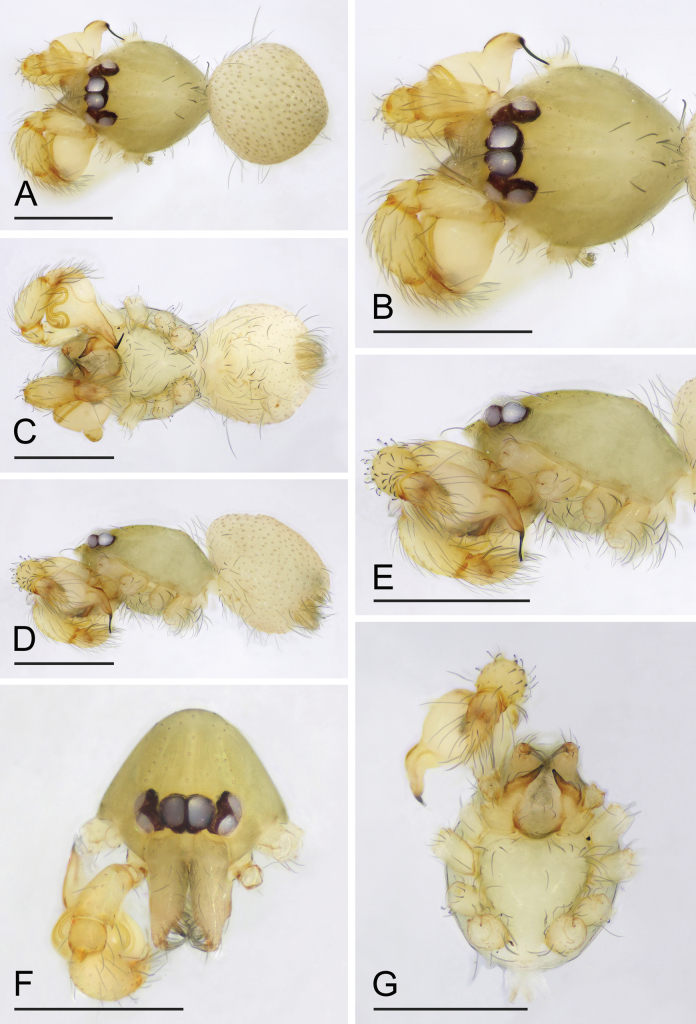
*Orchestinalini* sp. nov., holotype male **A, C, D** habitus, dorsal, ventral and lateral views **B, E, F, G** prosoma, dorsal, lateral, anterior and ventral views. Scale bars: 0.4 mm.

**Figure 2.**
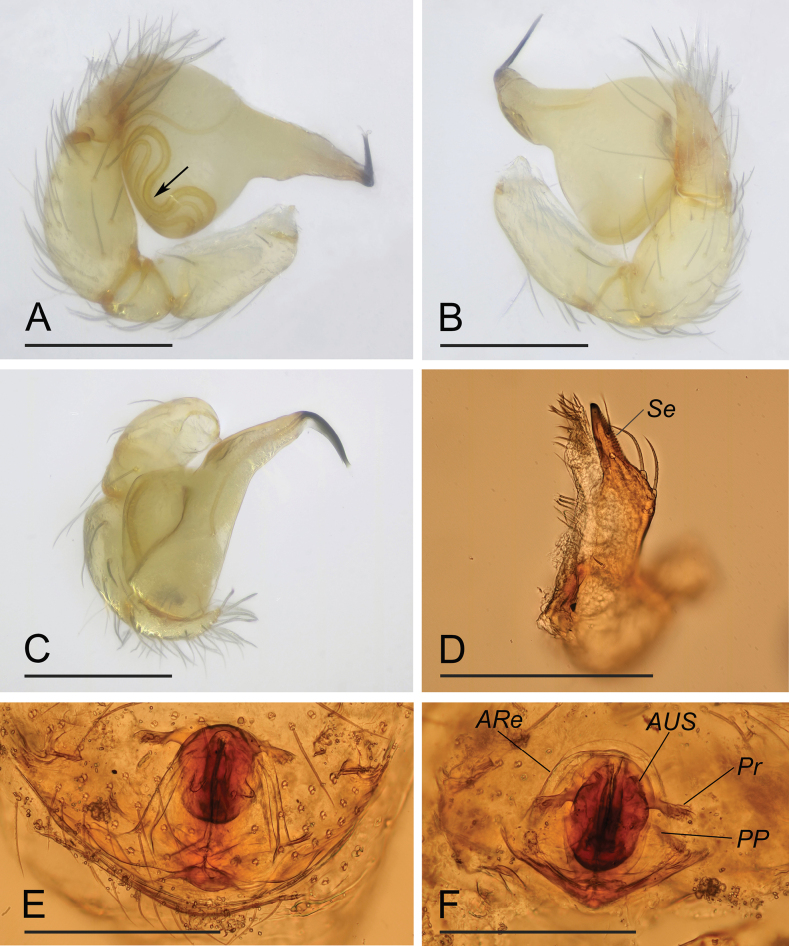
*Orchestinalini* sp. nov., holotype male (**A–D**), paratype female (**E, F**). **A–C** left palp, prolateral, retrolateral and dorsal views; arrow in **A** shows the abruptly bent **D** endites, ventral view **E, F** endogyne, ventral and dorsal views. Abbreviations: ARe = anterior receptaculum; AUS = anterior uterine sclerite; PP = posterior plate; Pr = protrusion; Se = serrula. Scale bars: 0.2 mm.

**Figure 3.**
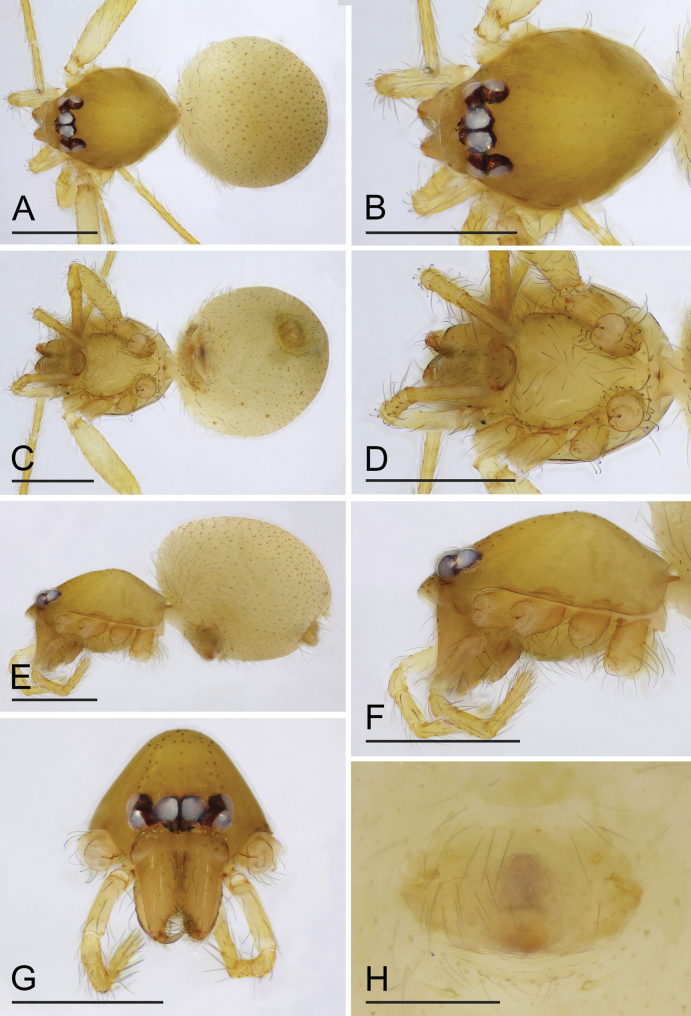
*Orchestinalini* sp. nov., paratype female **A, C, E** habitus, dorsal, ventral and lateral views **B, D, F, G** prosoma, dorsal, ventral, lateral and anterior views **H** epigaster, ventral view. Scale bars: 0.4 mm (**A–G**); 0.2 mm (**H**).

The new species is similar to *O.bialata* Liu, Xiao & Xu, 2016 in the shape of the bulb, but can be distinguished by the sperm duct abruptly bent in the middle section (Fig. 2A, arrow) vs. smoothly curved (Liu et al. 2019: fig. 7A), the round labium (Fig. 1G) vs. spade-shaped, with inverted Y-shaped sclerotized pattern (Liu et al. 2019: fig. 6B), and the epigaster without special external features (Fig. 3H) vs. with large triangular plate (Liu et al. 2016: fig. 5B, F). The new species is also similar to *O.clavulata* Tong & Li, 2011 in the female genitalia, but can be distinguished by the carapace without a net-shaped pattern (Figs 1A, 3A) vs. with a net-shaped pattern (Tong and Li 2011: fig. 1C, D).

#### Description.

**Male (holotype). *Body***: habitus as in Fig. 1A, C, D; body length 1.04. ***Carapace*** (Fig. 1B): 0.57 long, 0.46 wide; yellowish, oval in dorsal view, without net-shaped pattern, with some long setae, pars cephalica elevated in lateral view. ***Eyes*** (Fig. 1B, F): well developed, PME largest; posterior eye row recurved from above. ***Clypeus*** (Fig. 1E, F): margin unmodified, curved downwards in front view, sloping forward in lateral view. ***Sternum*** (Fig. 1G): longer than wide, surface smooth, setae sparse, needle-like, evenly scattered. ***Mouthparts*** (Figs 1F, G, 2D): chelicerae straight, anterior face unmodified; labium rounded, not fused to sternum, anterior margin not indented at middle; endites strongly sclerotized on outer margin, with serrula in single row. ***Abdomen*** (Fig. 1A, C, D): 0.49 long; ovoid, without net-shaped pattern. ***Legs***: yellow, without color pattern; femur IV thickened, wider than femora I−III. ***Palp*** (Fig. 2A–C): tibia slightly enlarged, length/width = 1.74, cymbium small; bulb pear-shaped in lateral view, with ventral side strongly protruding proximally, about 1.92 times as wide as tibia; the sperm duct abruptly bent in the middle section; embolus tapered, at almost right angle with long axis of bulbus.

**Female** (SYNU-F-090). Same as male except as noted. ***Body***: habitus as in Fig. 3A, C, E; body length 1.44. Carapace (Fig. 3B): 0.64 long, 0.49 wide. Abdomen: 0.82 long. ***Epigaster*** (Figs 2E, 3H): without special external features. ***Endogyne*** (Fig. 2F): with stout medial clavate sclerite (AUS), provided with pair of short lateral protrusions (Pr); anterior receptaculum (ARe) rounded, semitransparent, slightly longer than AUS; posterior plate (PP) present, large.

#### Distribution.

Known only from the type locality (Fig. 9).

### ﻿ Orchestina wuzu

Taxon classificationAnimaliaAraneaeOonopidae

Tong & Li sp. nov.

BB02E8AF-F9F7-55A8-87B5-9F56D5A8DF77

https://zoobank.org/4808D592-28AF-4224-8844-D9A29E4870F8

 Figs 4 –6

#### Material examined.

***Holotype*** China • ♂ (SYNU-F-056), ground searching; Xizang, Linzhi City, Nang Co., Dongga Town, Wuzu Vill.; 29°00.478'N, 93°13.547'E, 3062 ± 3 m; 11.VIII.2014; Y. Li leg. ***Paratypes*.** China • 20 ♂ 12 ♀ (SYNU-F-057–088), same data as holotype.

#### Etymology.

The specific name is a noun in apposition taken from the type locality.

#### Diagnosis.

**Figure 4.**
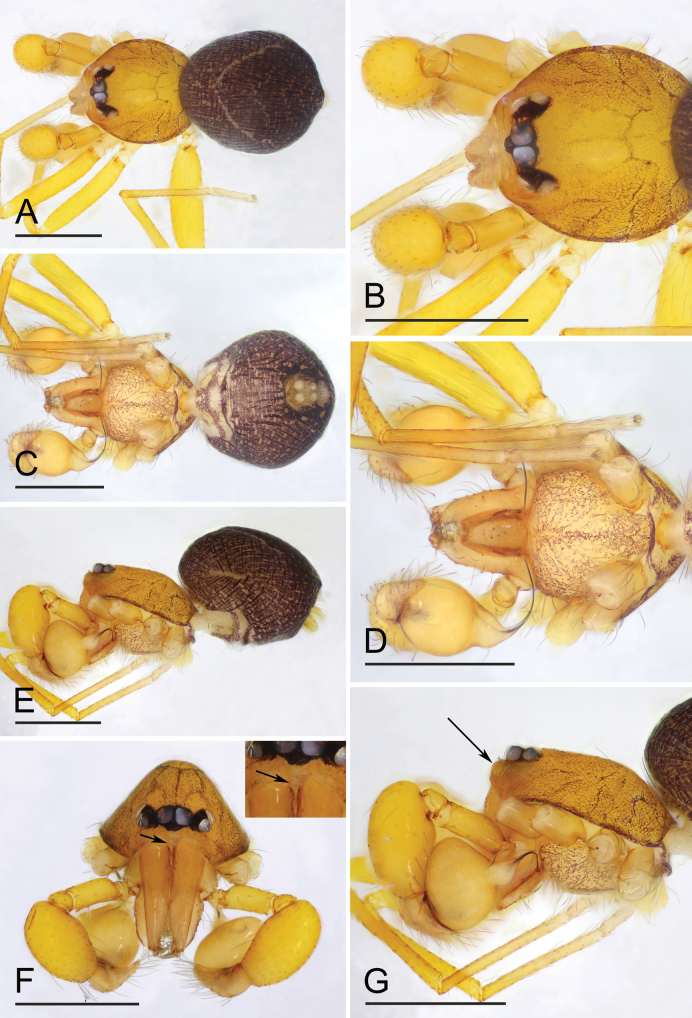
*Orchestinawuzu* sp. nov., holotype male **A, C, E** habitus, dorsal, ventral and lateral views **B, D, F, G** prosoma, dorsal, ventral, anterior and lateral views; arrow in **F** shows the median protrusion, arrow in **G** points to the forward clypeus. Scale bars: 0.4 mm.

**Figure 5.**
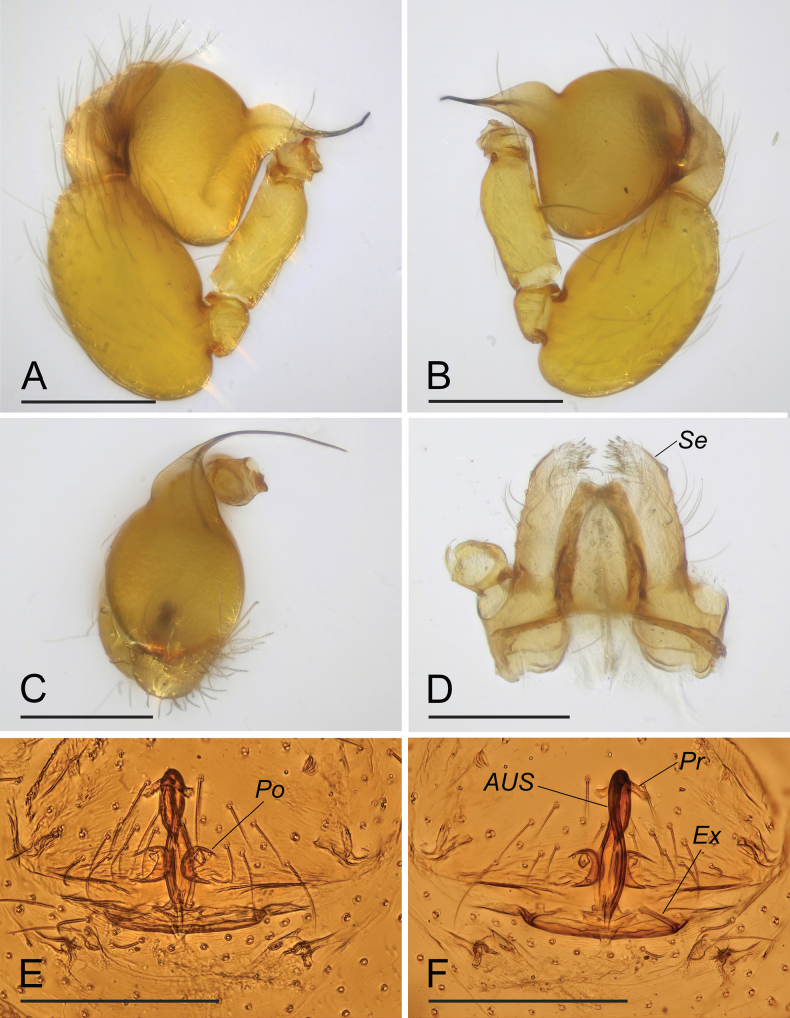
*Orchestinawuzu* sp. nov., holotype male (**A–D**), paratype female (**E, F**). **A–C** left palp, prolateral, retrolateral and dorsal views **D** endites and labium, ventral view **E, F** endogyne, ventral and dorsal views. Abbreviations: AUS = anterior uterine sclerite; Ex = dorsolateral extension; Po = pocket; Pr = protrusion; Se = serrula. Scale bars: 0.2 mm.

**Figure 6.**
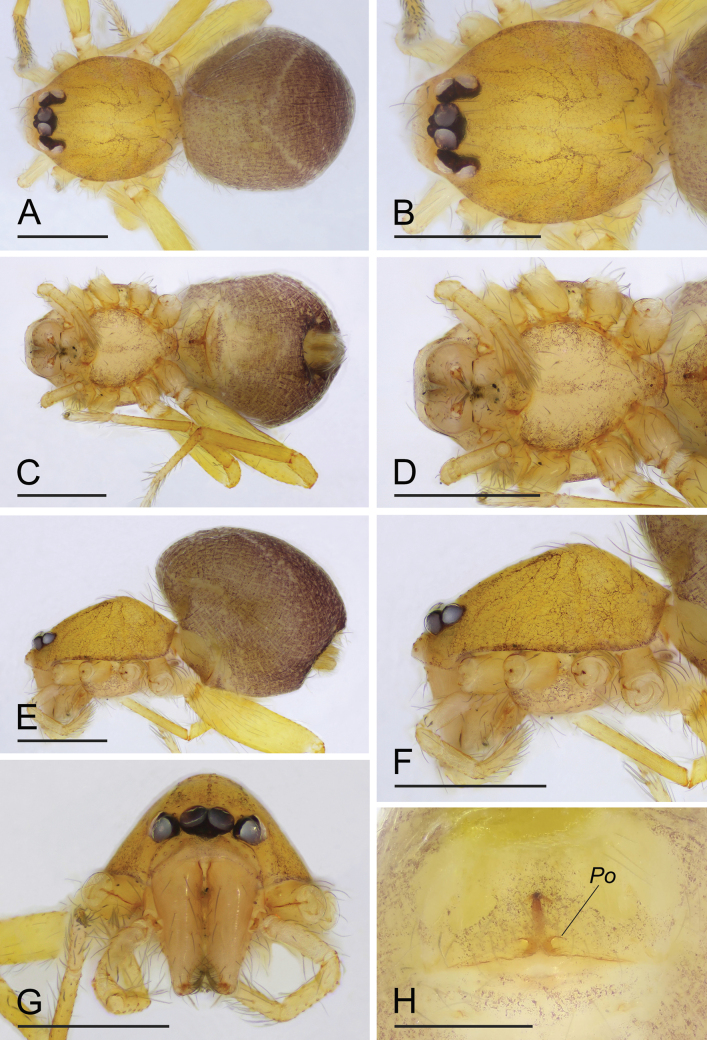
*Orchestinawuzu* sp. nov., paratype female **A, C, E** habitus, dorsal, ventral and lateral views **B, D, F, G** prosoma, dorsal, ventral, lateral and anterior views **H** epigaster, ventral view. Abbreviation: Po = pocket. Scale bars: 0.4 mm (**A–G**); 0.2 mm (**H**).

The new species is similar to *O.apiculata* Liu, Xiao & Xu, 2016, *O.dapojing* Tong & Yang, 2024 and *O.flagella* Saaristo & van Harten, 2006 in the shape of the bulb and the slender embolus, but can be distinguished by the median protrusion of male clypeus (Fig. 4F, arrow) vs. lacking (Saaristo and Van Harten 2006: fig. 52; Liu et al. 2016: fig. 1A, D; Wang et al. 2024a: fig. 1F), and the two small closely apposed semicircular sclerotized pockets (Figs 5E, F, 6H) vs. lacking in *O.apiculata* and *O.dapojing* (Liu et al. 2016: fig. 2C; Wang et al. 2024a: fig. 3G), and lacking, but with a glossy, banana-shaped transverse area in *O.flagella* (Saaristo and Van Harten 2006: fig. 54).

#### Description.

**Male (holotype). *Body***: habitus as in Fig. 4A, C, E; body length 1.12. ***Carapace*** (Fig. 4B): 0.50 long, 0.49 wide; orange brown, oval in dorsal view, with reticulate, net-shaped pattern, pars cephalica slightly curved in lateral view. ***Eyes*** (Fig. 4B, F): well developed, PME largest; posterior eye row recurved from above. ***Clypeus*** (Fig. 4F, G): curved downwards in front view, strongly sloping forward in lateral view; with a small median protrusion. ***Sternum*** (Fig. 4D): longer than wide, with dark brown patches; setae sparse, surface smooth. ***Mouthparts*** (Figs 4D, F, 5D): chelicerae straight and long, anterior face unmodified; labium elongated, not fused to sternum, anterior margin not indented at middle, lateral margins slightly sclerotized; endites unsclerotized, outer margin with serrula. ***Abdomen*** (Fig. 4A, C, E): 0.68 long; ovoid, with dense, gray net-shaped pattern and narrow pale chevron patterns. ***Legs***: yellow, without color pattern; femur IV thickened, wider than femora I−III. ***Palp*** (Fig. 5A–C): tibia of palp strongly enlarged, length/width = 1.75, cymbium small; bulb ovoid, about 1.33 times as wide as tibia, with distal part leading to embolus stout tube-shaped and inserted medially; the sperm duct barely visible through cuticle; embolus slender, whip-like, bent inwards.

**Female** (SYNU-F-058). Same as male except as noted. ***Body***: habitus as in Fig. 6A, C, E; body length 1.48. Carapace (Fig. 6B): 0.69 long, 0.56 wide. Chelicerae shorter than in males. Abdomen: 0.79 long. ***Epigaster*** (Figs 5E, 6H): basally with two small semicircular sclerotized pockets (Po), close to each other. ***Endogyne*** (Fig. 5F): with medial tubular sclerite (AUS), extending anteriorly, then flipped posteriorly, ending near epigastric furrow; anteriorly with two lateral protrusions (Pr); dorsolateral extension (Ex) interrupted anteriorly.

#### Distribution.

Known only from the type locality (Fig. 9).

### ﻿ Orchestina yigong

Taxon classificationAnimaliaAraneaeOonopidae

Tong & Li sp. nov.

1F8DB207-C0CA-5B31-AA9F-4FA0E29B1002

https://zoobank.org/F6DEEFB2-CE08-4AD2-B940-D20057C4BC2D

 Figs 7 , 8

#### Material examined.

***Holotype*** China • ♂ (SYNU-F-3702), sifting forest leaf litter; Xizang, Linzhi City, Pome Co., Yigong Town (S305 provincial highway 32 km away); 30°14.548'N, 94°51.303'E, 2175 m; 2.VIII.2017; M. Xu leg.

#### Etymology.

The specific name is a noun in apposition taken from the type locality.

#### Diagnosis.

**Figure 7.**
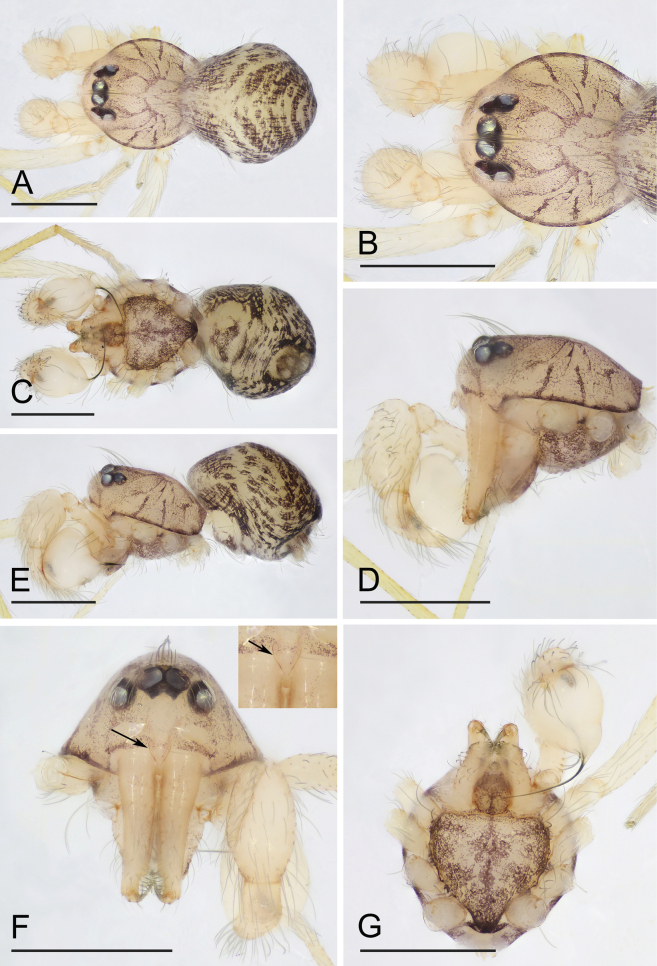
*Orchestinayigong* sp. nov., holotype male **A, C, E** habitus, dorsal, ventral and lateral views **B, D, F, G** prosoma, dorsal, lateral, anterior and ventral views, arrow in **F** shows the inverted drop-shaped protrusion. Scale bars: 0.4 mm (**A–G**).

**Figure 8.**
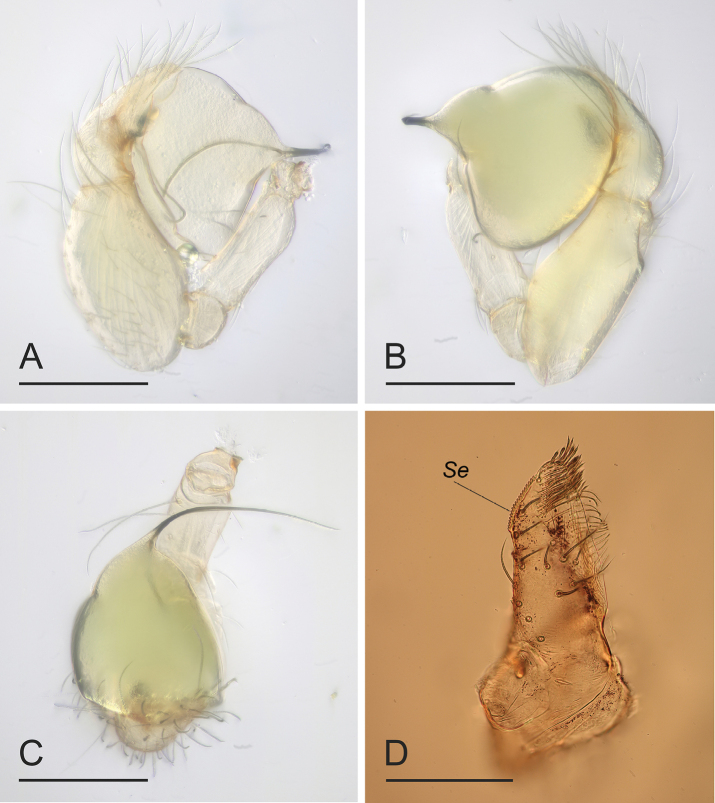
*Orchestinayigong* sp. nov., holotype male **A–C** left palp, prolateral, retrolateral and dorsal views **D** endites, ventral view. Abbreviation: Se = serrula. Scale bars: 0.2 mm.

The new species is similar to *O.apiculata* Liu, Xiao & Xu, 2016 and *O.dapojing* Tong & Yang, 2024 in the shape of the bulb and the long, whip-like embolus, but can be distinguished from *O.apiculata* by the carapace with a net-shaped pattern (Fig. 7A, B) vs. lacking (Liu et al. 2016: fig. 1A) and the palpal tibia narrower than the bulb (Fig. 8A, B) vs. distinctly wider than the bulb (Liu et al. 2016: fig. 2A, B); can be distinguished from *O.dapojing* by the clypeus with a small inverted drop-shaped protrusion (Fig. 7F) vs. lacking (Wang et al. 2024a: fig. 1A, B) and the long chelicerae (Fig. 7F) vs. very short (Wang et al. 2024a: fig. 1F).

#### Description.

**Male (holotype). *Body***: habitus as in Fig. 7A, C, E; body length 1.10. ***Carapace*** (Fig. 7B): 0.65 long, 0.52 wide; yellowish brown, oval in dorsal view, with net-shaped pattern, pars cephalica flat in lateral view. ***Eyes*** (Fig. 7B, F): well developed, nearly equal-sized; posterior eye row recurved from above. ***Clypeus*** (Fig. 7D, F): curved downwards in front view, sloping forward in lateral view; with a small inverted drop-shaped protrusion. ***Sternum*** (Fig. 7G): heart-shaped, with marginal band and median dark brown patches; setae sparse. ***Mouthparts*** (Figs 7F, G, 8D): chelicerae straight and long, anterior face unmodified; labium rounded, not fused to sternum, anterior margin not indented at middle; endites unsclerotized, outer margin with serrula. ***Abdomen*** (Fig. 7A, C, E): 0.65 long; ovoid, with gray net-shaped pattern and several broad chevron patterns. ***Legs***: yellow, without color pattern; femur IV thickened, wider than femora I−III. ***Palp*** (Fig. 8A–C): tibia strongly enlarged, length/width = 1.65, cymbium small; bulb pyriform, strongly enlarged, about 1.68 times as wide as tibia; the sperm duct thin, strongly curved; embolus nearly as long as bulb, whip-like, bent inwards.

**Female.** Unknown.

#### Distribution.

**Figure 9.**
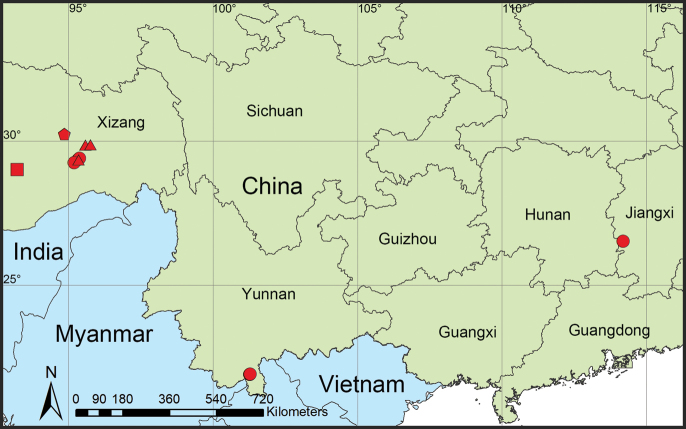
Distribution records of three new species and one known species of *Orchestina* from China. The circles represent *Orchestinacolubrina*, triangles represent *Orchestinalini* sp. nov., square represents *Orchestinawuzu* sp. nov., and the pentagon represents *Orchestinayigong* sp. nov.

Known only from the type locality (Fig. 9).

## ﻿Discussion

Sexual dimorphism is very common in the genus *Orchestina*. For example, the modified labial setae, endites with hook-shaped excrescences and rows of spectacular flattened setae, remarkable cheliceral setae and outgrowths, a modified clypeus, modified front legs, the preocular carapace swelling, and the infra-ocular gutter (Henrard and Jocqué 2012).

However, sexual modifications of the clypeus are usually associated with differences in shape; for example, the clypeus is sinuous, protruding, or backward in lateral view (Izquierdo and Ramírez 2017). Only two known species have a protrusion on the male clypeus, i.e., *O.lanceolata* Henrard & Jocqué, 2012, which has a rectangular sclerotized plate on the clypeus (Henrard and Jocqué 2012: figs 423, 434, 435); and *O.apiculata* has a small paddle-like protrusion on the clypeus, which was ignored in the original description, but can be seen in the original figures (Liu et al. 2016: fig. 3A, B). In the present study, *O.yigong* sp. nov. has a small inverted drop-shaped protrusion on the clypeus, and *O.wuzu* sp. nov. has a median protrusion on the clypeus, indicating variability of this trait.

## Supplementary Material

XML Treatment for Orchestina

XML Treatment for Orchestina colubrina

XML Treatment for Orchestina lini

XML Treatment for Orchestina wuzu

XML Treatment for Orchestina yigong

## ﻿Additional information

### Author ORCIDs

Yang Zhou https://orcid.org/0009-0007-1899-6503

Yanfeng Tong https://orcid.org/0000-0002-4348-7029

Dongju Bian https://orcid.org/0000-0002-9324-8026

Shuqiang Li https://orcid.org/0000-0002-3290-5416

### Data availability

All of the data that support the findings of this study are available in the main text.
